# Causal relationship between air pollution, lung function, gastroesophageal reflux disease, and non-alcoholic fatty liver disease: univariate and multivariate Mendelian randomization study

**DOI:** 10.3389/fpubh.2024.1368483

**Published:** 2024-04-29

**Authors:** Runmin Cao, Honghe Jiang, Yurun Zhang, Ying Guo, Weibin Zhang

**Affiliations:** ^1^Jinzhou Medical University Postgraduate Training Base (Jinzhou Central Hospital), Jinzhou, Liaoning, China; ^2^Department of Clinical Medicine, Anhui University of Science and Technology, Huainan, Anhui, China; ^3^Rehabilitation Therapy, Shandong Xiandai University, Jinan, Shandong, China; ^4^General Surgery, Jinzhou Central Hospital, Jinzhou, Liaoning, China

**Keywords:** air pollution, lung function, gastroesophageal reflux disease, non-alcoholic fatty liver disease, Mendelian randomization

## Abstract

**Background:**

The association between air pollution, lung function, gastroesophageal reflux disease, and Non-alcoholic fatty liver disease (NAFLD) remains inconclusive. Previous studies were not convincing due to confounding factors and reverse causality. We aim to investigate the causal relationship between air pollution, lung function, gastroesophageal reflux disease, and NAFLD using Mendelian randomization analysis.

**Methods:**

In this study, univariate Mendelian randomization analysis was conducted first. Subsequently, Steiger testing was performed to exclude the possibility of reverse association. Finally, significant risk factors identified from the univariate Mendelian analysis, as well as important factors affecting NAFLD from previous observational studies (type 2 diabetes and body mass index), were included in the multivariable Mendelian randomization analysis.

**Results:**

The results of the univariable Mendelian randomization analysis showed a positive correlation between particulate matter 2.5, gastroesophageal reflux disease, and NAFLD. There was a negative correlation between forced expiratory volume in 1 s, forced vital capacity, and NAFLD. The multivariable Mendelian randomization analysis indicated a direct causal relationship between gastroesophageal reflux disease (OR = 1.537, *p* = 0.011), type 2 diabetes (OR = 1.261, *p* < 0.001), and NAFLD.

**Conclusion:**

This Mendelian randomization study confirmed the causal relationships between air pollution, lung function, gastroesophageal reflux, and NAFLD. Furthermore, gastroesophageal reflux and type 2 diabetes were identified as independent risk factors for NAFLD, having a direct causal connection with the occurrence of NAFLD.

## 1 Introduction

In recent years, non-alcoholic fatty liver disease (NAFLD) has become a global public health challenge, with an increase in incidence and its association with various metabolic disorders (1, 2). As a result, research and prevention of NAFLD have become a focal point in contemporary medical field. However, the pathogenesis of NAFLD remains incompletely understood to date. In recent years, researchers have increasingly focused on the association between air pollution, decline in lung function, and gastroesophageal reflux disease (GERD) with NAFLD.

The intensifying air pollution in modern industrialized societies has become a significant global public health issue. Previous studies have found environmental pollutants increase the risk of NAFLD (3). Researchers speculate on the following mechanisms that could lead to this result: Firstly, fine particulate matter can cause insulin resistance (IR) through endothelial dysfunction, affecting the liver insulin signaling pathway, inhibiting the expression of peroxisome proliferator-activated receptor (PPAR) γ and PPARα, leading to hepatic lipid accumulation (4). Secondly, fine particulate matter can activate Kuppfer cells by promoting the expression of pro-inflammatory factors in adipocytes, leading to NAFLD (5). Additionally, fine particulate matter can further promote the development of NAFLD by affecting endoplasmic reticulum stress, oxidative stress, gut environment, and microRNA expression (6). However, Li et al.’s (7) study found no association between particulate matter 2.5 (PM2.5) concentration and liver fat measurements. Therefore, further research is needed to determine the causal relationship between air pollution and NAFLD.

Furthermore, numerous studies have demonstrated an association between decreased lung function and the development of NAFLD (8–10). Additionally, a study in the UK found a negative correlation between lung function and insulin resistance (11). Given the close association between NAFLD and insulin resistance, decreased lung function may increase the risk of NAFLD by promoting insulin resistance (8). However, the majority of current studies on the association between lung function and NAFLD are from Asian countries. Although the National Health and Nutrition Examination Survey III (NHANES-III) study has shown an independent correlation between NAFLD and decreased lung function in American adults, the generalizability of these findings to European populations still requires further validation (9).

Finally, GERD is a common gastrointestinal condition. A meta-analysis found that patients with GERD have a significantly increased risk of developing NAFLD (12). Elevated levels of cytokines and chemokines in the serum of GERD patients, as well as activation of oxidative stress, may play a crucial role in the development of NAFLD (13–15). However, it is currently unclear whether this association is causal or the result of shared underlying risk factors (12).

The aforementioned studies on the relationship between air pollution, lung function, GERD, and the risk of NAFLD primarily rely on cross-sectional and case–control studies, which can only describe the correlation between risk factors and the target outcome, without being able to determine a causal relationship (16). Mendelian randomization (MR), as an emerging causal inference method in genetic epidemiology, can reduce biases resulting from confounding factors and reverse causation in traditional observational studies, thereby enabling a more accurate evaluation of the causal relationship between exposure and outcome (17). Multivariable Mendelian randomization (MVMR) is a novel technique that integrates genetic variations related to multiple risk factors into a single model, allowing for the simultaneous evaluation of multiple exposures while minimizing the influence of confounding variables (18). Accordingly, we used a large-scale genome-wide association study dataset to conduct univariable Mendelian randomization (UVMR) and MVMR analyses, delving into whether there is a causal relationship between air pollution, decreased lung function, GERD, and NAFLD. These findings aim to provide more precise scientific evidence for future preventive and therapeutic strategies for NAFLD.

## 2 Methods

### 2.1 Data sources

Our study utilized summary datasets from genome-wide association studies (GWAS) conducted in Europe. Data on air pollution indicators, including PM2.5, PM2.5–10, PM10, nitrogen oxides, and nitrogen dioxide, were sourced from the UK Biobank and accessed through the MRC IEU OpenGWAS platform (19).^1^ As for lung function indicators, they include forced vital capacity (FVC) and forced expiratory volume in 1 s (FEV1). For the analysis of FVC, we utilized GWAS data from the UK Biobank cohort, obtained through the BOLT-LMM Bayesian mixed model association method by Loh et al. (20) which encompassed a total of 422,876 participants. The research data on FEV1 originated from the UK Biobank and included 345,665 participants. We used the largest GWAS dataset on gastroesophageal reflux disease currently available in the European population, comprising a total sample size of 602,604, with 129,080 cases and 473,524 controls. Further details on this GWAS data can be found in the study by Ong et al. (21).The genetic association with body mass index (BMI) was extracted from a GWAS study by the Genetic Investigation of ANthropometric Traits (GIANT) consortium. The study encompassed 125 investigations involving 339,224 individuals, including 322,154 of European ancestry and 17,072 of non-European ancestry, and we selected the GWAS data of the European population from this study (22). The dataset for type 2 diabetes was derived from a meta-analysis by Sakaue et al., which incorporated GWAS summary results from three population-based projects, namely, the Biobank Japan (BBJ), UK Biobank, and FinnGen. The meta-analysis included a total of 490,089 individuals of European ancestry and 177,415 individuals of East Asian ancestry, and we employed GWAS data from individuals of European ancestry for our study (23).

The outcome data were obtained from FinnGen, a notable research collaboration between public and private entities. This groundbreaking project merges estimated genotype data from newly collected and legacy samples originating from the Finnish Biobank, along with digital health records data obtained from the Finnish National Health Registers. The integration of these extensive datasets offers a unique and innovative perspective on disease genetics, unraveling novel insights in the field (24). We obtained the GWAS dataset for NAFLD with the GWAS ID from FinnGen version 9, which included 2,275 cases and 375,002 controls, and this dataset consisted entirely of individuals of European descent. The specific details of the GWAS datasets mentioned above can be found in Supplementary Table S1.

### 2.2 Selection of genetic instrumental variables

To conduct the MR analysis effectively and reliably, we employed the following selection criteria for instrumental variables (IVs): firstly, we set the statistical significance threshold at the genome-wide significance level of *p* < 5 × 10–8. Secondly, using a reference panel of European population genotypes from the 1,000 Genomes Project, we calculated the linkage disequilibrium (LD) between SNPs of each risk factor. SNPs in LD (R2 > 0.001, within a 10,000 kb window) were excluded, retaining the SNP with the lowest *p*-value. Furthermore, we excluded palindromic SNPs with ambiguous minor allele frequencies falling outside the range of >0.45– < 0.55, as well as incompatible allele combinations from different individuals at two or more loci, to ensure the integrity of the dataset (25). We also calculated the F statistic, F = beta^2/SE^2, to assess the strength of the IVs, excluding those with corresponding F-statistic values below 10 as weak IVs (26). Additionally, we performed leave-one-out sensitivity analysis, excluding one SNP at a time and conducting IVW analysis on the remaining SNPs to exclude specific SNPs driving significant effects (27). Finally, to reduce heterogeneity and avoid pleiotropic effects, we utilized the MR Pleiotropy Residual Sum and Outlier (MR-PRESSO) method, which employs residual-based outlier detection, to identify and exclude significant levels of pleiotropic outliers that may confound the results.

### 2.3 Statistical analysis

We employed a Mendelian randomization (MR) analysis approach, utilizing summary statistics from exposure and outcome GWAS datasets from different countries, to estimate causal effects and enhance the statistical power and accuracy of the MR analysis. We employed three MR methods, including weighted median regression, inverse-variance weighted (IVW), and Mendelian randomization-Egger (MR-Egger) methods, to evaluate the associations between air pollution, lung function, GERD, and the risk of NAFLD incidence. The IVW method was the primary approach for the MR analysis. The MR-Egger intercept represents the average pleiotropic effect of the genetic instruments, while the slope coefficient accounts for directional imbalance and provides a valid estimate of the causal effect (28). We also conducted a global test using MR-PRESSO to detect the presence of pleiotropic outliers and adjusted the causal estimates after removing any outliers, if detected. Furthermore, we performed Cochran’s Q test to assess heterogeneity among the instruments (29). We conducted MR Steiger directionality tests to refute biases caused by reverse causation (30).

**Figure 1:**
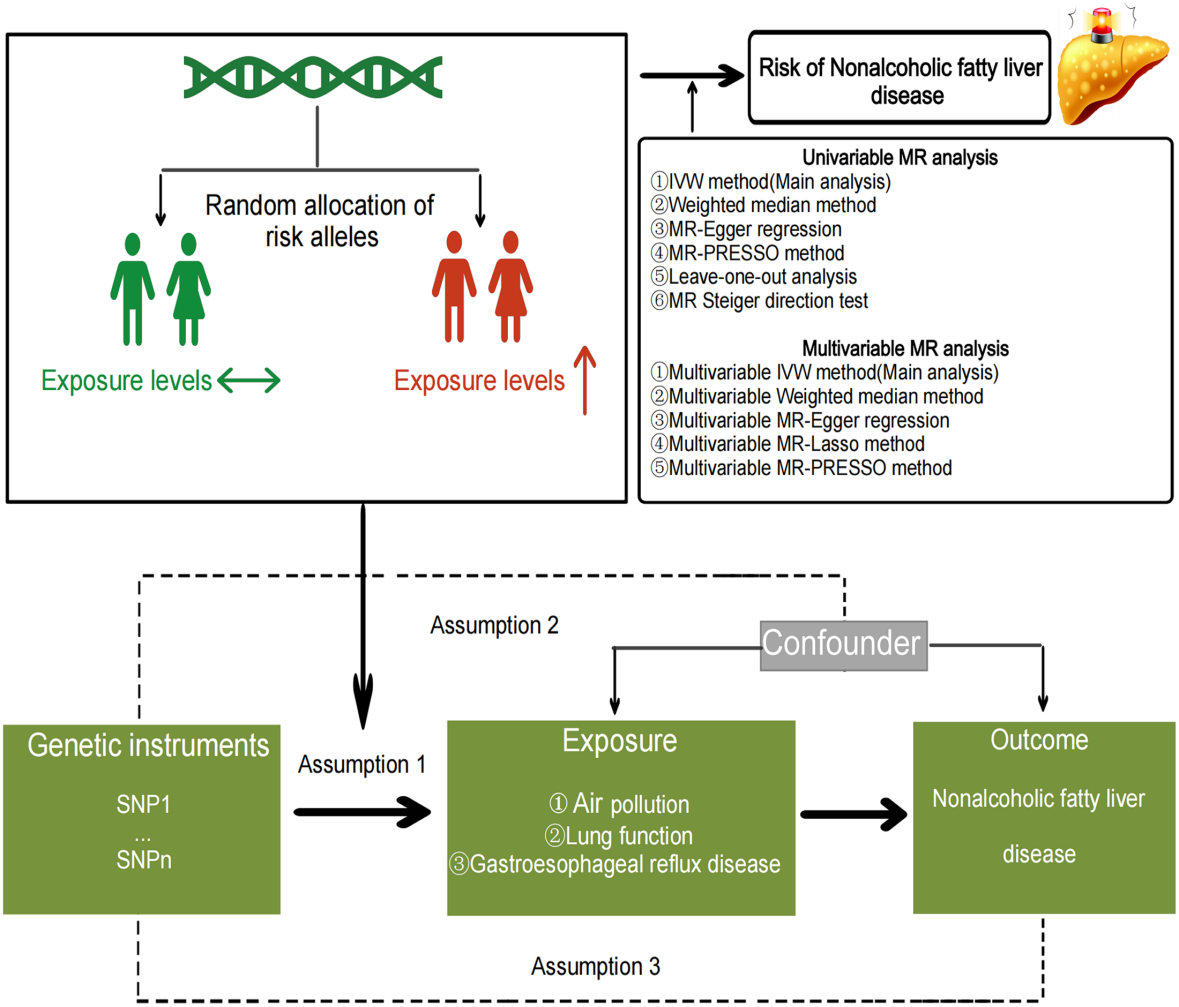
The specific flow chart of the study.

Previous observational studies have suggested that obesity and type 2 diabetes are important risk factors for the occurrence of NAFLD (31, 32). Therefore, in our multivariable MR analysis, we included key risk factors identified in the univariable Mendelian randomization analysis, as well as BMI and type 2 diabetes, aiming to identify independent risk factors influencing non-alcoholic liver disease. We utilized MVMR-IVW, MVMR-Median, and MVMR-Egger to assess the independent associations of each exposure factor with the outcome, with MVMR-IVW serving as the primary analytical method (29). Furthermore, the multivariable MR least absolute shrinkage and selection operator (LASSO) method was employed to evaluate the presence of collinearity among multiple exposure factors, and if collinearity was detected, the MVMR results were adjusted accordingly (33). MVMR-PRESSO global test and MVMR-Egger intercept test were used to detect residual pleiotropy (29). Mendelian randomization statistical analysis and data visualization were performed using the “MendelianRandomization,” “TwoSampleMR,” “MRPRESSO,” and “MVMR” packages in R software (version 4.2.2), and the flowchart of the study is depicted in Figure 1. Bonferroni correction was applied to adjust *p*-values based on the number of exposures. Associations with two-sided *p*-values <0.007 (= 0.05/7 exposures) were considered statistically significant, while associations with two-sided *p*-values <0.05 were considered suggestive.

## 3 Results

### 3.1 Genetic instrumental variable

In the GWAS, we identified a large number of independent SNPs (*p* < 5 × 10–8) associated with the following traits: 75 SNPs associated with gastroesophageal reflux disease, 8 SNPs associated with PM2.5, 21 SNPs associated with PM10um, 4 SNPs associated with Nitrogen dioxide, 8 SNPs associated with Nitrogen oxides, 211 SNPs associated with FEV1, and 319 SNPs associated with FVC (Table 1). However, no independent SNPs were identified for PM2.5–10 below the threshold of *p* < 5 × 10–8. Leave-one-out sensitivity analysis revealed specific SNPs, rs114789974 and rs77205736, with significant driving effects in PM10 and Nitrogen dioxide, respectively. After removing these specific SNPs, the leave-one-out analysis showed no SNPs with significant influence after sequentially excluding any single SNP (Supplementary Figure S1). Furthermore, the F statistics for all included independent SNPs in the study were above the threshold of 10, indicating low evidence for weak instrument bias in this study (detailed data on the IVs can be found in Supplementary Table S2).

**Table 1:** Univariate Mendel randomized results of air pollution, lung function, GERD and NAFLD.

Exposure	Number of SNPs	Mendelian randomization method	OR(95% CI)	*p* value
PM2.5	8	Inverse-variance weighted	3.445 (1.073,11.061)	0.038*
	8	Weighted median	3.096 (1.046,9.163)	0.041*
	8	Mendelian randomization-Egger	3.297 (0.637,17.055)	0.205
PM10um	21	Inverse-variance weighted	1.473 (0.443,4.902)	0.528
	21	Weighted median	1.933 (0.377,9.923)	0.430
	21	Mendelian randomization-Egger	4.755 (0.149,152.023)	0.389
Nitrogen dioxide	4	Inverse-variance weighted	2.7057 (0.139,52.708)	0.511
	4	Weighted median	3.566 (0.108,117.617)	0.476
	4	Mendelian randomization-Egger	0.454 (0.000,46598.161)	0.906
Nitrogen oxides	8	Inverse-variance weighted	0.399 (0.054,2.927)	0.366
	8	Weighted median	0.491 (0.035,6.794)	0.596
	8	Mendelian randomization-Egger	1.858 (0.000,382246.900)	0.924
FEV1	211	Inverse-variance weighted	0.561 (0.411,0.766)	<0.001**
	211	Weighted median	0.568 (0.363,0.891)	0.014*
	211	Mendelian randomization-Egger	0.325 (0.121,0.876)	0.027*
FVC	319	Inverse-variance weighted	0.637 (0.517,0.785)	<0.001**
	319	Weighted median	0.688 (0.507,0.934)	0.016*
	319	Mendelian randomization-Egger	0.546 (0.264,1.129)	0.103
GERD	75	Inverse-variance weighted	1.544 (1.218,1.956)	<0.001**
	75	Weighted median	1.526 (1.099,2.118)	0.012*
	75	Mendelian randomization-Egger	1.074 (0.265,4.357)	0.920

*p < 0.05; **p < 0.007.

PM2.5, particulate matter 2.5; PM10, particulate matter 10; FEV1, Forced expiratory volume in 1-s; FVC, Forced Vital Capacity; GERD, gastroesophageal reflux disease.

### 3.2 Univariate Mendelian randomized analysis results

**Figure 2:**
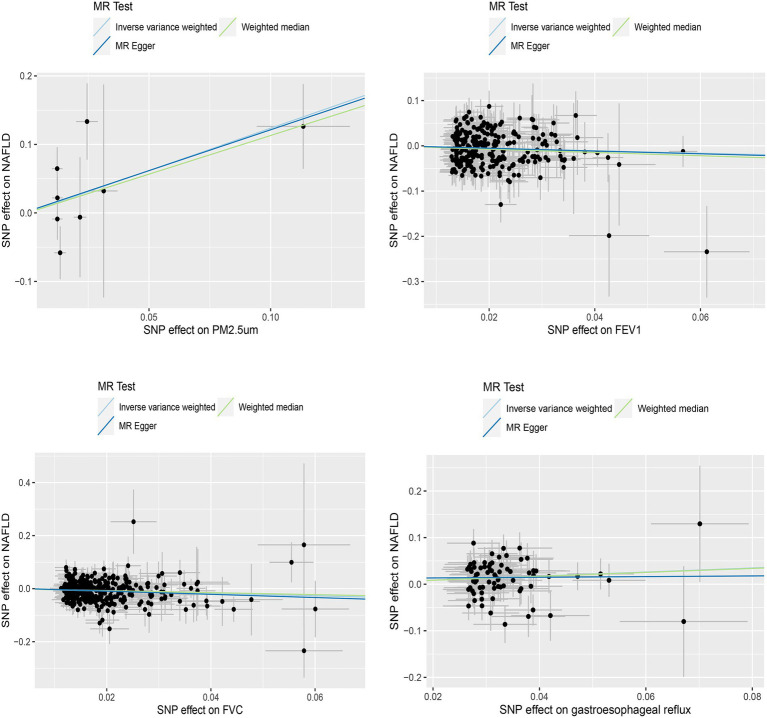
Scatterplot of significantly associated (IVW derived *p* < 0.05) and directionally consistent estimates.

We initially conducted univariable Mendelian randomized (UVMR) analyses using SNPs associated with the exposures of interest. In the IVW analysis, genetically predicted GERD (OR = 1.544 [1.218, 1.956], *p* < 0.001) and higher levels of PM2.5 (OR = 3.445 [1.073, 11.061], *p* = 0.038) were associated with an increased risk of NAFLD, whereas lower FEV1 (OR = 0.561 [0.411, 0.766], *p* < 0.001) and FVC (OR = 0.637 [0.517, 0.785], *p* < 0.001) were associated with an increased risk of developing NAFLD (Table 1). Furthermore, the IVW, MR-Egger, and weighted median estimates showed consistent directions of effect (Figure 2). We did not observe any causal relationships between PM10 (OR = 1.473 [0.443, 4.902], *p* = 0.528), Nitrogen dioxide (OR = 2.706 [0.139, 52.708], *p* = 0.511), Nitrogen oxides (OR = 0.399 [0.054, 2.927], *p* = 0.366), and NAFLD (Table 1). In the MR Steiger test, we did not find evidence of reverse causality in the analysis (Table 2). In univariable MR analyses, all Cochran’s Q statistics had *p*-values greater than 0.05, indicating no heterogeneity. Both the MR-Egger intercept test and the MR-PRESSO global test were not statistically significant (*p* > 0.05), indicating no evidence of horizontal pleiotropy (Table 2).

**Table 2:** Univariable Mendelian analysis sensitivity analysis results.

Exposure	Mendelian randomization Egger_Q	Mendelian randomization Egger_Q_pval	Inverse-variance weighted_Q	Inverse-variance weighted_Q_pval	Pleiotropy_pval	Mendelian randomizationPRESSO_pval	Steiger_pval	Correct_causal_direction
PM2.5	10.544	0.104	10.556	0.159	0.938	0.377	<0.001*	True
PM10 um	23.224	0.228	23.837	0.25	0.487	0.244	<0.001*	True
Nitrogen dioxide	0.091	0.955	0.19	0.979	0.783	0.981	<0.001*	True
Nitrogen oxides	4.434	0.618	4.496	0.721	0.811	0.715	<0.001*	True
FEV1	240.786	0.065	242.274	0.063	0.257	0.084	<0.001*	True
FVC	299.105	0.757	299.294	0.767	0.664	0.758	<0.001*	True
GERD	79.114	0.292	79.401	0.313	0.608	0.237	<0.001*	True

*p < 0.05.

PM2.5, particulate matter 2.5; PM10, particulate matter 10; FEV1, Forced expiratory volume in 1-s; FVC, Forced Vital Capacity; GERD, gastroesophageal reflux disease.

### 3.3 Multivariate Mendelian randomized analysis results

**Figure 3:**
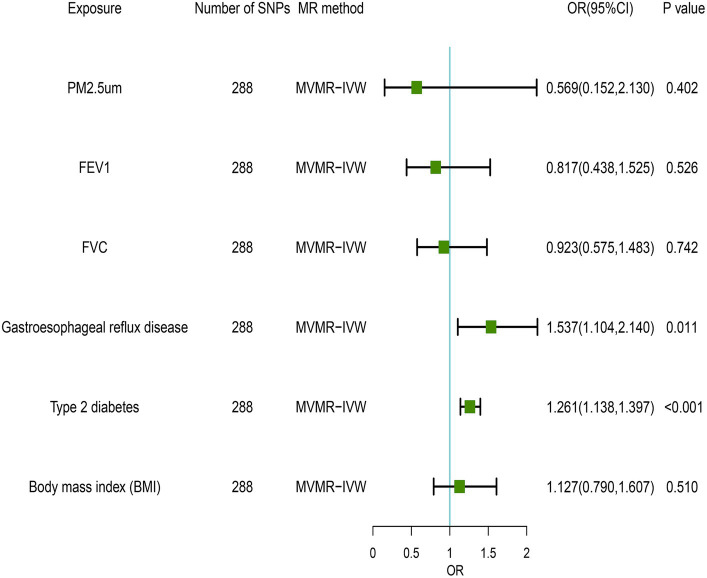
Multivariate Mendelian randomization results of air pollution, lung function, gastroesophageal reflus disease, diabetes, BMI and Non-alcoholic liver disease.

In the MVMR analysis, we evaluated the relationships between GERD, PM2.5, FEV1, FVC, type 2 diabetes, BMI, and NAFLD. We conducted a lasso test and found no evidence of potential collinearity issues. The results of the MVMR analysis revealed that GERD (OR = 1.537 [1.104, 2.140], *p* = 0.011) and type 2 diabetes (OR = 1.261 [1.138, 1.397], *p* < 0.001) were independent risk factors for NAFLD (Figure 3). This suggests that GERD and type 2 diabetes are direct risk factors for NAFLD, with GERD directly increasing the risk of NAFLD by 0.537 times and type 2 diabetes directly increasing the risk by 0.261 times. Meanwhile, PM2.5, FEV1, and FVC impact the incidence of NAFLD through mediation by independent factors. The MVMR-Egger intercept test (*p* = 0.079) and MVMR-PRESSO global test (*p* = 0.405) did not detect horizontal pleiotropy. Moreover, no heterogeneity was observed in the MVMR-IVW (*p* = 0.376) and MVMR-Egger (*p* = 0.410) methods (Table 3).

**Table 3:** Supplementary and sensitivity analyses for the causal relationship between NAFLD and multiple factors.

Exposure	Number of SNPs	Mendelian randomization method	OR(95%CI)	*p* value	Mendelian randomization-Egger intercept test *p* value	Mendelian randomization-PRESSO global test *p* value
PM2.5 um	288	Multivariable Mendelian randomization-Median	0.285 (0.126, 0.596)	0.129	0.410	0.405
	288	Multivariable Mendelian randomization-Egger	0.531 (0.099, 2.242)	0.347		
FEV1	288	Multivariable Mendelian randomization-Median	1.177 (0.483, 1.995)	0.709		
	288	Multivariable Mendelian randomization-Egger	0.799 (0.392, 1.470)	0.478		
FVC	288	Multivariable Mendelian randomization-Median	0.814 (0.510, 1.288)	0.536		
	288	Multivariable Mendelian randomization-Egger	0.946 (0.705, 1.181)	0.820		
GERD	288	Multivariable Mendelian randomization-Median	1.277 (0.850, 2.124)	0.263		
	288	Multivariable Mendelian randomization-Egger	1.995 (1.279, 3.127)	0.002**		
Type 2 diabetes	288	Multivariable Mendelian randomization-Median	1.262 (1.093, 1.439)	0.006**		
	288	Multivariable Mendelian randomization-Egger	1.258 (1.137, 1.393)	<0.001**		
BMI	288	Multivariable Mendelian randomization-Median	1.403 (0.924, 2.609)	0.174		
	288	Multivariable Mendelian randomization-Egger	1.122 (0.767, 1.507)	0.523		

*p < 0.05; **p < 0.007.

PM2.5, particulate matter 2.5; FEV1, Forced expiratory volume in 1-s; FVC, Forced Vital Capacity; GERD, gastroesophageal reflux disease; BMI, Body Mass Index.

## 4 Discussion

### 4.1 Main findings

In the univariable MR analysis, we found that GERD, higher levels of PM2.5, and lower FEV1 and FVC were associated with an increased risk of NAFLD. The multivariate MR results indicated that only GERD and type 2 diabetes were independent factors for NAFLD, with a direct causal relationship. The effects of PM2.5, FEV1, and FVC on NAFLD were masked by independent factors, suggesting that PM2.5, FEV1, and FVC may indirectly influence the incidence of NAFLD through mediating the independent factors.

### 4.2 The role of GERD in NAFLD

Our study revealed a direct causal relationship between GERD and an increased risk of NAFLD. This is consistent with some previous research findings. A meta-analysis showed that individuals with GERD had approximately twice the risk of developing NAFLD compared to those without GERD (12). A case–control study conducted in Poland also found a higher susceptibility of NAFLD among patients with GERD (34). GERD may promote the development of NAFLD through multiple mechanisms. Firstly, serum levels of cytokines and chemokines, such as tumor necrosis factor-α, and interleukin-1β, are elevated in GERD patients (13). These factors contribute to the recruitment and activation of Kupffer cells, leading to an inflammatory response in non-alcoholic steatohepatitis (14). Secondly, oxidative stress could be another plausible explanation for the higher incidence of NAFLD in patients with GERD. GERD is associated with increased levels of free radicals and peroxynitrite in the esophageal mucosa, both of which play important roles in driving non-alcoholic steatohepatitis (15, 35). Additionally, platelet activation factors produced and released by the esophageal mucosa in GERD may play a significant role in linking GERD and NAFLD. These activation factors, by inducing the release of other inflammatory mediators and stimulating hepatic lipid synthesis, contribute to the association between GERD and NAFLD (36, 37). However, there is currently limited research on the association between GERD and NAFLD, necessitating larger-scale epidemiological studies to further support our conclusions.

### 4.3 Impact of PM2.5 exposure and lung function on NAFLD risk

The results of this study indicate that elevated levels of PM2.5 and decreased lung function are risk factors for NAFLD. A study involving 99,556 individuals confirmed our hypothesis that levels of PM2.5 are positively associated with NAFLD (38). This finding is further supported by experimental studies conducted in mice (39). In our univariable Mendelian randomization analysis, PM2.5 was found to increase the risk of NAFLD by 2.445 times. However, in the multivariable Mendelian randomization analysis, PM2.5 was not identified as an independent risk factor for NAFLD. These results suggest that the association between PM2.5 exposure and NAFLD risk may be mediated by other factors. Consistent with this, a cross-sectional analysis conducted in 45 states in the United States revealed that the association between PM2.5 exposure and the risk of NAFLD was influenced by age, race/ethnicity, diabetes, smoking, and geographical region (40). In this study, univariable Mendelian randomization analysis revealed that the decrease in FEV1 and FVC is associated with an increased risk of NAFLD. However, multivariable Mendelian randomization analysis showed that FEV1 and FVC are not independent risk factors for NAFLD. This suggests that FEV1 and FVC may influence the risk of NAFLD through other factors. A cross-sectional study demonstrated that insulin resistance partly mediates the relationship between FVC, FEV1, and NAFLD, which further supports our research findings (41). Previous studies have shown that decreased lung function is associated with elevated levels of inflammatory markers, which are believed to promote the development of type 2 diabetes and consequently increase the risk of NAFLD (42–44).

### 4.4 The association between type 2 diabetes, BMI and NAFLD

In our study, we found that type 2 diabetes was also directly associated with an increased risk of NAFLD, which is supported by previous research providing etiological explanations for this association. Huang et al. conducted a multicenter study and found that high insulin resistance, a key pathological feature of type 2 diabetes, was considered the most important predictor of NAFLD, regardless of whether the subjects were obese or lean (45). The main reason is that insulin resistance leads to the accumulation of fat in the liver, and during the process of fat accumulation in the liver, cellular damage and insulin resistance further exacerbate liver inflammation and fibrosis (46, 47). In contrast to some previous observational studies, our study results suggest that BMI is not a direct factor leading to NAFLD. This discrepancy may be attributed to the smaller sample sizes and uncontrolled potential variables in those studies (48, 49). The research findings of Yuan et al. corroborate our conclusion that type 2 diabetes might be a key mediator in the pathway from BMI to NAFLD (50). Specifically, this may be due to the fact that pancreatic beta cells are the only cells that produce insulin, but in individuals with higher BMI, beta-cell function is impaired and pancreatic volume is reduced, thereby promoting the development of type 2 diabetes and further increasing the risk of NAFLD incidence (51–53).

### 4.5 Strengths and limitations of the study

According to our knowledge, this is the first MR study to estimate the genetic causal relationships between air pollution, lung function, GERD, and NAFLD. This MR study has several notable strengths. Firstly, we selected SNPs that were significantly associated with air pollution, lung function, and GERD, while excluding the influence of weak SNPs. Secondly, we conducted directionality tests to exclude the impact of reverse causality on our study results. Thirdly, we performed multiple sensitivity analyses in both univariable and multivariable Mendelian randomization analyses to verify the robustness of these findings. Lastly, we employed multivariable Mendelian randomization analysis to incorporate two important influencing factors identified in observational studies (type 2 diabetes and BMI) to assess the independent effects of NAFLD. However, our study still has some limitations. Firstly, due to the use of summary-level data from GWAS databases, we were unable to evaluate the non-linear correlations between air pollution, lung function, GERD, and NAFLD. Secondly, air pollution and lung function vary greatly throughout a person’s life, but our MR analysis estimated lifetime exposure to air pollution and lung function as genetic variations are fixed at conception. It cannot assess the relationship between air pollution, lung function at different stages of life and the risk of NAFLD, which requires longitudinal cohort studies to comprehensively explore this association. Additionally, given the limited number of genetic instruments for air pollution, the accuracy of statistical models may be affected by high standard errors, suggesting the need for larger-scale GWAS data to further validate our conclusions. Finally, the participants in this study were of European ancestry, which, while reducing population stratification bias, limits the generalizability of our findings to other populations.

## 5 Conclusion

In summary, this study elucidates the causal relationships between PM2.5, lung function, and GERD with NAFLD, with GERD being identified as an independent risk factor for NAFLD. These findings have implications for clinicians, suggesting the need for increased vigilance regarding the presence of NAFLD in individuals living in highly polluted areas, with reduced lung function or suffering from GERD. Additionally, this study provided clues to the potential mechanisms by which the increased concentration of PM2.5 and decreased lung function lead to an increased risk of NAFLD, suggesting that PM2.5 and lung function may indirectly promote the occurrence of NAFLD by influencing the development of type 2 diabetes. Given the current lack of sufficient research on the relationship between air pollution, lung function, GERD, and NAFLD, more large-scale studies are needed to validate our findings and further explore the potential causal mechanisms.

## Data availability statement

The original contributions presented in the study are included in the article/Supplementary material, further inquiries can be directed to the corresponding author.

## Ethics statement

The requirement of ethical approval was waived by Jinzhou central hospital ethics committee for the studies involving humans because ethical approval and written informed consent were provided in the original publications and these publicly available databases. The studies were conducted in accordance with the local legislation and institutional requirements. The ethics committee/institutional review board also waived the requirement of written informed consent for participation from the participants or the participants’ legal guardians/next of kin because ethical approval and written informed consent were provided in the original publications and these publicly available databases.

## Author contributions

RC: Conceptualization, Data curation, Methodology, Software, Visualization, Writing – original draft. HJ: Conceptualization, Methodology, Visualization, Writing – original draft. YZ: Methodology, Visualization, Writing – original draft. YG: Visualization, Writing – original draft. WZ: Writing – review & editing.

## Footnotes

^1^ https://gwas.mrcieu.ac.uk/
