# Supplementary figures and images for: Causal relationship between air pollution, lung function, gastroesophageal reflux disease, and non-alcoholic fatty liver disease: univariate and multivariate Mendelian randomization study
Source: Front Public Health. 2024 Apr 29;12:1368483. doi: 10.3389/fpubh.2024.1368483 (PMC11092889; doi:10.3389/fpubh.2024.1368483)

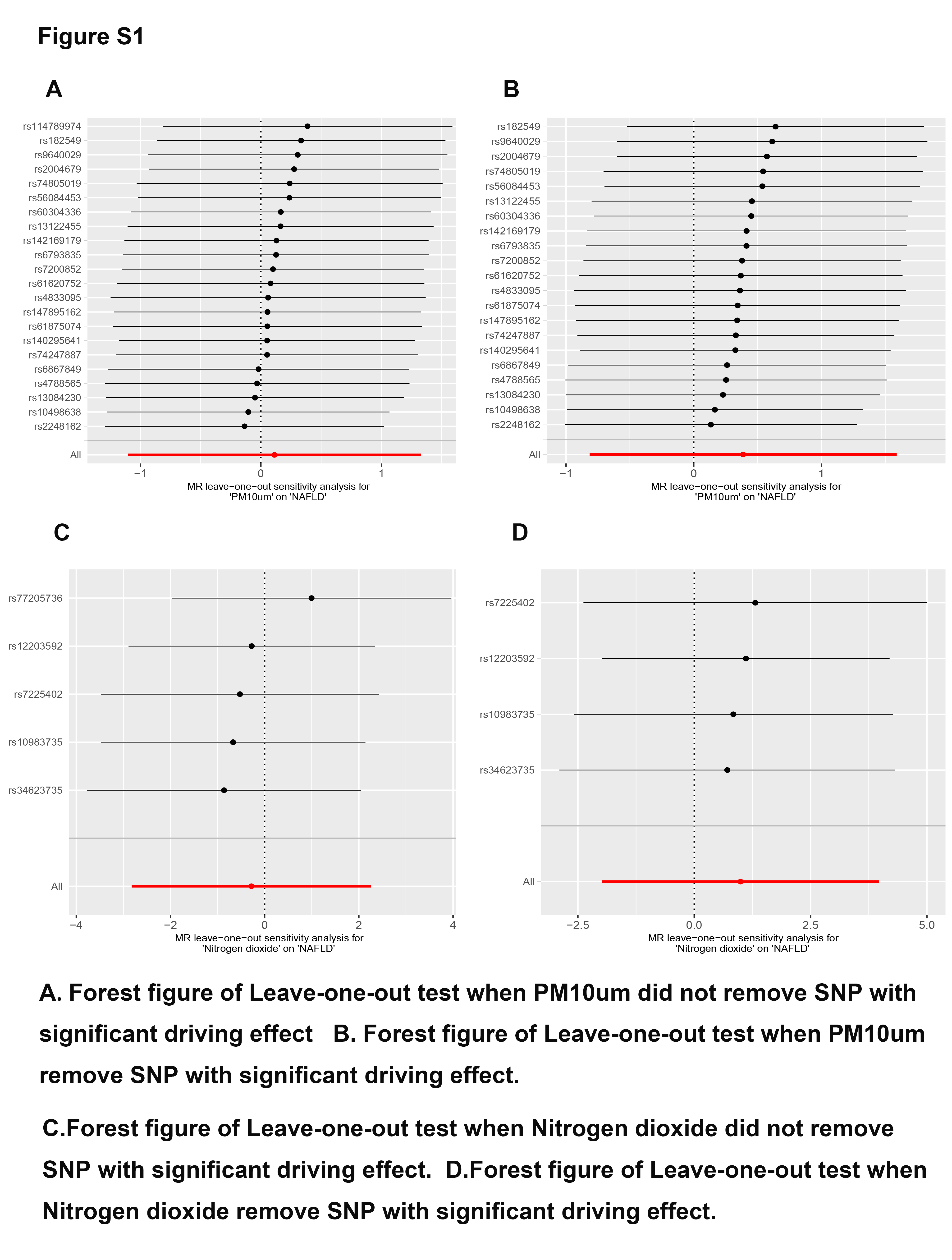

Supplement: Supplementary file 3 [file Image_1.tif]
